# Supplementary material for: Complementary cognitive roles for D2-MSNs and D1-MSNs during interval timing
Source: eLife. 2025 Jan 15;13:RP96287. doi: 10.7554/eLife.96287 (PMC11735027; doi:10.7554/eLife.96287)
Supplement: Supplementary file 1. [file elife-96287-supp1.docx]

| **Condition** | **Source** | **α** | **β** |  |
| --- | --- | --- | --- | --- |
| D2-MSNs Laser Off | Empirical Data | 6.03 | 0.67 | Figure 4 - figure supplement 3A |
| D2-MSNs Laser Off | Model | 6.08 | 0.69 | Figure 4 - figure supplement 3E |
| D2-MSNs Inhibited | Empirical Data | 7.62 | 0.78 | Figure 4 - figure supplement 3B |
| D2-MSNs Inhibited | Model | 7.67 | 0.79 | Figure 4 - figure supplement 3F |
| D1-MSNs Laser Off | Empirical Data | 5.87 | 0.68 | Figure 4 - figure supplement 3C |
| D1-MSNs Laser Off | Model | 5.89 | 0.69 | Figure 4 - figure supplement 3G |
| D1-MSNs Inhibited | Empirical Data | 7.13 | 0.70 | Figure 4 - figure supplement 3D |
| D1-MSNs Inhibited | Model | 7.31 | 0.72 | Figure 4 - figure supplement 3H |
